# Development and evaluation of the Newstage system: integrating tumor regression grade and lymph node status for improved prognostication in neoadjuvant treatment of gastric cancer

**DOI:** 10.1186/s12957-023-03291-4

**Published:** 2024-01-10

**Authors:** Ming Chen, Shanshan Yu, Cheng Chen, Jinxiao Liang, Donghui Zhou

**Affiliations:** Department of Surgical Oncology, The First Affiliated Hospital, School of Medicine, Zhejiang University, Hangzhou, China

**Keywords:** Neoadjuvant treatment, Cancer staging, Tumor regression grade, Prognosis, Gastric cancer

## Abstract

**Background:**

The predictive correlation of tumor depth of invasion changes after neoadjuvant therapy, and the 8th American Joint Committee on Cancer (AJCC) ypTNM system for gastric cancer may not accurately predict patient prognosis following neoadjuvant therapy.

**Methods:**

A retrospective analysis was conducted on a total of 258 patients who underwent radical surgery for gastric cancer after neoadjuvant therapy. The Newstage system was established based on tumor regression grade and pathological lymph node status. The 3-year survival rates of patients classified by the Newstage system were compared with those classified by the AJCC ypTNM system.

**Results:**

In a cohort of 258 patients, the 3-year overall survival rates based on the Newstage system were: (I) 94.6%, (II) 79.3%, (III) 54.5%, and (IV) 30.2%. The Newstage system exhibited a lower Akaike information criterion value (902.57 vs. 912.03). Additionally, the area under the ROC curve (0.756 vs. 0.733) and the C-index (0.731 vs. 0.718) was higher than the AJCC ypTNM system. Furthermore, a multivariate analysis indicated that the Newstage system was an independent prognostic factor (*p* = 0.001).

**Conclusion:**

The Newstage system exhibits superior predictive performance in estimating survival rates for neoadjuvant therapy in gastric cancer. It also functions as an independent prognostic factor.

**Supplementary Information:**

The online version contains supplementary material available at 10.1186/s12957-023-03291-4.

## Introduction

Gastric cancer is a common type of tumor in the digestive tract, ranking fourth in both incidence and mortality according to the 2020 Global Cancer Statistics [1]. According to the 2016 Cancer Report released by the National Cancer Center of China in 2022, gastric cancer ranks third in both incidence and mortality in China [2]. Neoadjuvant therapy, based on clinical trials such as MAGIC [3], CLASSIC [4], and RESOLVE [5], is now the standard treatment for advanced gastric cancer, improving overall survival for patients. The 8th edition of the American Joint Committee on Cancer (AJCC) has incorporated survival analysis using prospective data collected by the national cancer database in collaboration with the International Cancer Society. This collaboration aims to enhance the staging of post-neoadjuvant therapy for gastric cancer patients by implementing the ypTNM staging system. This provides crucial guidance to clinicians in making informed decisions throughout the treatment process [6]. After receiving neoadjuvant therapy, tumors have the potential to undergo shrinkage, necrosis, and degeneration. The depth of infiltration, which is an important factor in staging the disease, can be influenced by the variability in treatment response. This may not fully reflect the treatment effect and prognosis. Tumor regression grade (TRG) is an important prognostic factor in gastrointestinal tumors [7, 8]. However, a favorable tumor regression does not necessarily lead to a change in T staging. Therefore, the aim of this study is to establish a Newstage system based on TRG and ypN staging and compare its prognostic accuracy with the AJCC ypTNM system.

## Methods

### Patients and treatment

This retrospective cohort analysis included a total of 258 patients with gastric cancer who underwent neoadjuvant therapy followed by radical resection (R0 resection) at the First Affiliated Hospital of Zhejiang University School of Medicine between January 1, 2015, and July 31, 2020. The inclusion criteria for the study were as follows: histologically confirmed gastric adenocarcinoma, receipt of neoadjuvant or conversion therapy before surgery with complete removal of the tumor. Exclusion criteria encompassed incomplete histopathological or survival data, residual gastric cancer, the presence of diffuse distant metastasis, death within 30 days after surgery, and palliative resection. Detailed clinical, surgical, and postoperative pathological information was collected, including age, gender, TRG, T stage, N stage, tumor size, tumor location, tumor differentiation, preoperative chemotherapy regimen, utilization of combined immunotherapy, postoperative adjuvant chemotherapy, date of death, and validation through telephone follow-up or electronic medical records. TRG grading was determined by two specialized pathologists based on a retrospective review of the available pathological specimens. The staging system utilized in the analysis was based on the AJCC 8th edition guideline [9]: TRG0 (complete response), no viable cancer cells, including nodes; TRG1 (near complete response), single cells or rare small groups of cancer cells; TRG2 (partial response), residual cancer cells with evident tumor regression but more than single cells or rare small groups of cancer cells; TRG3 (poor or no response), extensive residual cancer with no evident tumor regression.

### Statistics

The statistical analysis using R software version 4.1.3. The TRG grades were grouped with ypN stages into several combinations, and integrated the 3-year overall survival (OS) data with the combination of TRG and ypN status. The best fit combination that could describe prognosis and survival was chosen to become the Newstage system. We compared survival curves using the Kaplan–Meier method and log-rank test. Correlations were performed using Spearman’s correlation and expressed by Spearman’s coefficient. Using univariate and multivariate Cox regression models, we selected prognostic variables associated with OS. For the Cox multivariate analysis, we used a two-step multivariate approach. In the first step of the multivariate analysis, all significantly important prognostic factors from the univariate analysis (*p* < 0.05) as well as common risk factors were included, excluding the Newstage system. In the second step of the multivariate analysis, the Newstage system was added to all the factors included in the first step. We generated time-dependent receiver operating characteristic (ROC) curves and calculated the estimated area under the curve (AUC) to compare the prognostic ability of two staging systems. We used the R package “timeROC” for this analysis. We compared the AUC of AJCC ypTNM and the Newstage system using AUC estimators. We performed internal validation using the bootstrap method and evaluated model performance through repeated iterations. We also assessed the relative discriminatory ability of the two staging systems using the Akaike information criterion (AIC) and Harrell’s concordance index (C-index). A lower AIC indicates a better fit, while a higher C-index suggests better discriminatory ability. We considered differences significant at *p* < 0.05 in two-sided tests.

## Results

### Clinical and pathological data

Among the 258 patients, 69.8% were male (*n* = 180), and 67.1% of the patients were over 60 years old. Regarding the selection of neoadjuvant treatment, 68.2% of the patients underwent the SOX chemotherapy regimen (*n* = 176), followed by XELOX (*n* = 46). In the postoperative pathological analysis, the most common tumor regression grade according to AJCC was TRG2 (*n* = 88, 34.1%). In the AJCC 8th edition ypTNM staging, ypStage III was the most prevalent stage (*n* = 78, 30.2%), and the most common occurrence of lymph node metastasis was ypN0 (*n* = 139, 53.9%). The proportion of patients achieving pathological complete response (pCR) or ypT0N + was 12.8% (*n* = 33). Furthermore, the majority of tumors were located in the lower part of the stomach (50.4%, *n* = 130), with a size of ≤ 5 cm (81.8%, *n* = 211) and poor differentiation (48.8%, *n* = 126). Most patients proceeded with postoperative adjuvant chemotherapy (*n* = 222, 86.0%), 35.6% of the patients (*n* = 79) received SOX chemotherapy regimen, while other regimens included paclitaxel plus S-1, oxaliplatin, irinotecan, and docetaxel. Please refer to Table 1 for specific details.

**Table 1:** Clinical and pathological data for patients associated with ypN status

	ypN0 *N* = 139	ypN1 *N* = 50	ypN2 *N* = 32	ypN3a *N* = 25	ypN3b *N* = 12	*p*.overall
Gender						0.886
F	40 (28.8%)	16 (32.0%)	9 (28.1%)	8 (32.0%)	5 (41.7%)	
M	99 (71.2%)	34 (68.0%)	23 (71.9%)	17 (68.0%)	7 (58.3%)	
Age						0.778
≤ 60	44 (31.7%)	17 (34.0%)	10 (31.2%)	8 (32.0%)	6 (50.0%)	
> 60	95 (68.3%)	33 (66.0%)	22 (68.8%)	17 (68.0%)	6 (50.0%)	
TRG						
0	32 (23.0%)	4 (8.00%)	1 (3.12%)	0 (0.00%)	0 (0.00%)	
1	39 (28.1%)	4 (8.00%)	3 (9.38%)	0 (0.00%)	2 (16.7%)	
2	38 (27.3%)	24 (48.0%)	10 (31.2%)	11 (44.0%)	5 (41.7%)	
3	30 (21.6%)	18 (36.0%)	18 (56.2%)	14 (56.0%)	5 (41.7%)	
ypT						
T0	32 (23.0%)	4 (8.00%)	1 (3.12%)	0 (0.00%)	0 (0.00%)	
T1	28 (20.1%)	9 (18.0%)	1 (3.12%)	0 (0.00%)	0 (0.00%)	
T2	31 (22.3%)	6 (12.0%)	4 (12.5%)	1 (4.00%)	0 (0.00%)	
T3	32 (23.0%)	20 (40.0%)	19 (59.4%)	13 (52.0%)	2 (16.7%)	
T4a	15 (10.8%)	11 (22.0%)	6 (18.8%)	11 (44.0%)	9 (75.0%)	
T4b	1 (0.72%)	0 (0.00%)	1 (3.12%)	0 (0.00%)	1 (8.33%)	
ypStage						
pCR	28 (20.1%)	0 (0.00%)	0 (0.00%)	0 (0.00%)	0 (0.00%)	
ypT0N +	0 (0.00%)	4 (8.00%)	1 (3.12%)	0 (0.00%)	0 (0.00%)	
ypStage I	62 (44.6%)	9 (18.0%)	0 (0.00%)	0 (0.00%)	0 (0.00%)	
ypStage II	47 (33.8%)	24 (48.0%)	5 (15.6%)	0 (0.00%)	0 (0.00%)	
ypStage III	2 (1.44%)	13 (26.0%)	26 (81.2%)	25 (100%)	12 (100%)	
Size(cm)						< 0.001
≤ 5	128 (92.1%)	43 (86.0%)	23 (71.9%)	14 (56.0%)	3 (25.0%)	
> 5	11 (7.91%)	7 (14.0%)	9 (28.1%)	11 (44.0%)	9 (75.0%)	
Location						
Diffuse	3 (2.16%)	1 (2.00%)	1 (3.12%)	0 (0.00%)	1 (8.33%)	
Lower	71 (51.1%)	26 (52.0%)	16 (50.0%)	11 (44.0%)	6 (50.0%)	
Middle	35 (25.2%)	11 (22.0%)	11 (34.4%)	12 (48.0%)	5 (41.7%)	
Upper	30 (21.6%)	12 (24.0%)	4 (12.5%)	2 (8.00%)	0 (0.00%)	
Differentiation						
High	5 (3.60%)	2 (4.00%)	0 (0.00%)	0 (0.00%)	0 (0.00%)	
Middle	49 (35.3%)	21 (42.0%)	16 (50.0%)	8 (32.0%)	1 (8.33%)	
Poor	61 (43.9%)	24 (48.0%)	15 (46.9%)	15 (60.0%)	11 (91.7%)	
Unknown	24 (17.3%)	3 (6.00%)	1 (3.12%)	2 (8.00%)	0 (0.00%)	
Neoadjuvant						
SOX	99 (71.2%)	34 (68.0%)	25 (78.1%)	13 (52.0%)	5 (41.7%)	
XELOX	25 (18.0%)	8 (16.0%)	3 (9.38%)	5 (20.0%)	5 (41.7%)	
FOLFOX	7 (5.04%)	6 (12.0%)	3 (9.38%)	3 (12.0%)	2 (16.7%)	
ELSE	8 (5.76%)	2 (4.00%)	1 (3.12%)	4 (16.0%)	0 (0.00%)	
Post-operative adjuvant therapy						0.740
Yes	118 (84.9%)	43 (86.0%)	30 (93.8%)	21 (84.0%)	10 (83.3%)	
No	21 (15.1%)	7 (14.0%)	2 (6.25%)	4 (16.0%)	2 (16.7%)	
Combined immunotherapy						0.175
Yes	24 (17.3%)	6 (12.0%)	5 (15.6%)	6 (24.0%)	5 (41.7%)	
No	115 (82.7%)	44 (88.0%)	27 (84.4%)	19 (76.0%)	7 (58.3%)	

Data are median (IQR) or *n* (%), *SOX* S-1 and oxaliplation; *XELOX* capecitabine and oxaliplatin; *FOLFOX* fluorouracil, leucovorin, oxaliplatin; *ELSE* including infrequent chemotherapy regimens such as SPA, DCF, and FOLT

We determined the correlation between the TRG and ypT stage. Spearman’s correlation coefficient (ρ) values and *P* value for the assessment of statistical significance are presented in Table 2. According to the Spearman correlation analysis, the Spearman rank correlation coefficient between TRG and ypT is 0.675, indicating a moderate positive correlation between TRG and ypT staging, which is statistically significant (*p* < 0.001).

**Table 2:** Correlations between the TRG and ypT

Characteristic	Spearman’s ρ	*p* value
ypT	0.675	< 0.001

## Outcomes

**Fig. 1:**
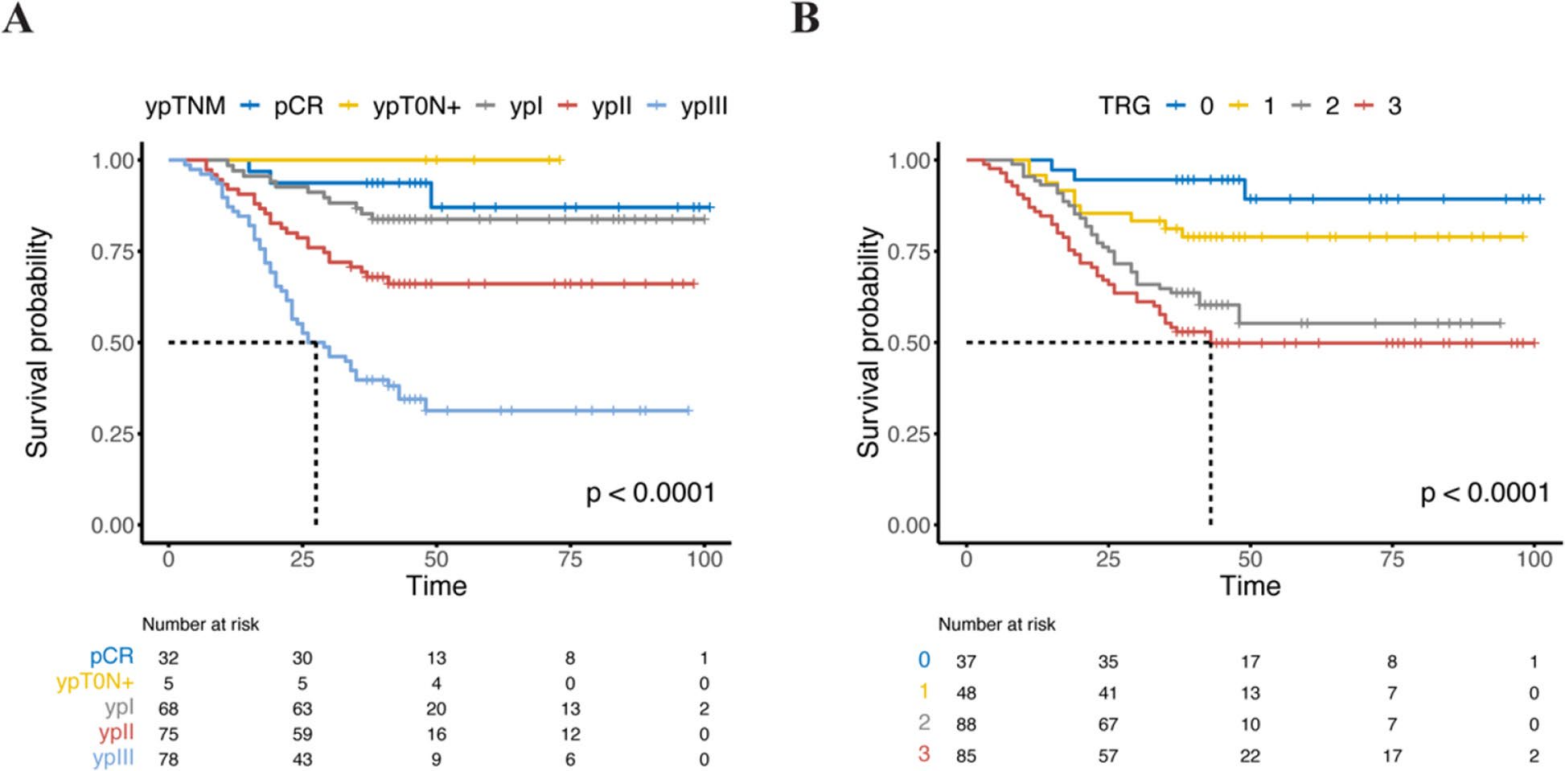
Kaplan–Meier overall survival according to the 8th AJCC ypTNM stage (**A**), and AJCC TRG gradings (**B**)

The median follow-up was 41 months. The 3-year overall survival (OS) rate 68%. To the 8th AJCC ypTNM staging system, the 3-year OS rate for patients with stage pCR was 93%, ypT0N + was 100%; stage ypStage I was 86%; for stage ypStage II, 70%; and for stage ypStage III, 40% with statistically significant differences among all stages (*p* < 0.001, Fig. 1A). The 3-year survival rates of ypStage I are similar to those of pCR and ypT0N + stages, as well as the survival rates of ypStage II, which are similar to the ypT0N + stage (*p* > 0.05, Supplementary Table S1). The difference between ypT0N + and ypStage II is not statistically significant, which may be related to the 5 cases in the ypT0N + group. To the 8th AJCC pTNM staging system, the 3-year OS rate for patients with stage pCR and pT0N + were consistent with the above stages. For pStage IA–pStage IIIC were 86%, 77%, 71%, 69%, 50%, 32%, and 8% (*p* < 0.001, Supplementary Figure S1A). Three-year overall survival was compared between each of the pTNM stage groupings, the differences between stages within the pTNM were not particularly pronounced (Supplementary Table S2). The survival curves, stratified by the AJCC TRG grading and ypN status, are depicted in Fig. 1B and Supplementary Figure S1B. Although the survival curves of the four staging methods mentioned above all show differences, it is worth noting that ypN status also demonstrates a noticeable trend of separation in terms of survival outcomes.

## Newstage system

**Fig. 2:**
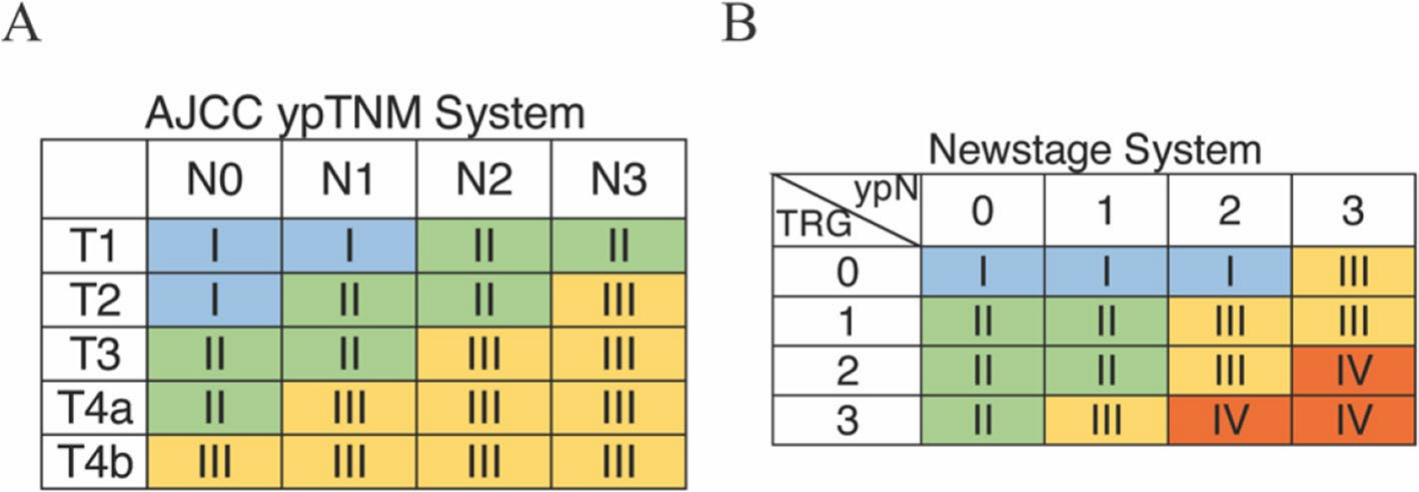
AJCC ypTNM stage system (**A**) and Newstage system (**B**)

A novel staging system was devised by integrating the 3-year overall survival (OS) data with the combination of TRG and ypN status (Supplementary Table S3). The survival curves, stratified by the combination of TRG and ypN status, are depicted in Supplementary Figure S2. The Newstage system was compared with the AJCC ypTNM stage system in Fig. 2.

**Fig. 3:**
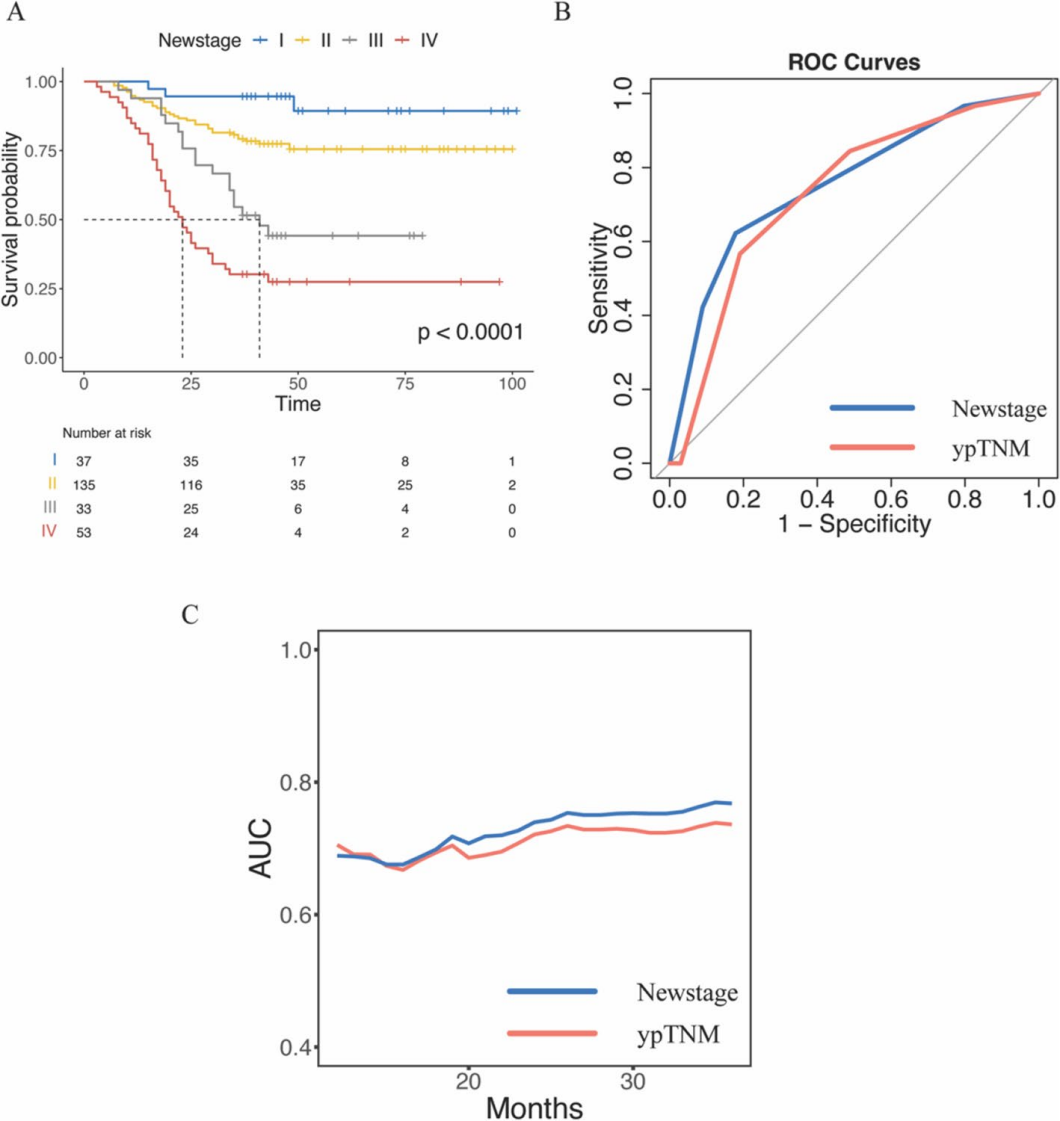
Kaplan–Meier overall survival according to Newstage system (**A**). Comparing the ROC curves of the ypTNM stage system and the Newstage system (**B**). Time-dependent ROC curves for ypTNM system and Newstage systems (**C**). Solid lines represent time-dependent ROC curves

The Newstage demonstrated more concordant 3-year survival (Newstage I, 94.6%; Newstage II, 79.3%; Newstage III 54.5%; Newstage IV 30.2%). The survival curves showed better separation although all stages showed significant differences (*p* < 0.001, Fig. 3A). Three-year overall survival was compared between each of the Newstage substages (*p* < 0.005, Supplementary Table S4).

### Comparison of the AJCC ypTNM and Newstage system

The integration of estimated areas under the ROC curves demonstrated that the Newstage system outperformed the ypTNM stage system in predicting 3-year overall survival, with an AUC of 0.756 compared to 0.733 (Fig. 3B). Internal validation using the Bootstrap method with 2000 resamples further supported the superiority of the Newstage system, as evidenced by the mean AUC of 0.756 (Supplementary Figure S3). Additionally, the time-dependent ROC curve analysis revealed that the Newstage system had a superior performance compared to the ypTNM stage system (Fig. 3C). Furthermore, the Newstage system exhibited a smaller Akaike Information Criteria (AIC) value (902.57 vs. 912.03), indicating a better fit to the data. The C-index, which measures the concordance between predicted and observed outcomes, was also higher for the Newstage system compared to the ypTNM stage system (0.731 vs. 0.718). These findings collectively suggest that the Newstage system offers more precise prognostic stratification and improved predictive accuracy compared to the ypTNM stage system.

### Univariable and multivariable analysis

To investigate the prognostic assessment capabilities of different staging systems, we conducted a two-step multivariate Cox analysis (Table 3). In step 1, the multivariate analysis included significant prognostic factors from univariate Cox analysis as well as common risk factors, with the exclusion of the Newstage system. The variables considered encompassed age, tumor size, differentiation type, TRG, tumor location, neoadjuvant regimens, and the AJCC ypTNM stage system. Within this step, ypTNM stage emerged as an independent prognostic factor for OS (*p* < 0.001). In step 2, the multivariate analysis comprised all factors from Step 1, while introducing the Newstage system. The findings indicated that the Newstage system and Differentiation were independent prognostic factors for OS (*p* = 0.001, *p* = 0.007), whereas the ypStage system no longer demonstrated significance (*p* = 0.190).

**Table 3:** Two-step multivariate analysis for survival using Cox regression analysis on cohort

Characteristic	HR^a^	95% CI^a^	*p* value
Step 1			
Age	1.38	0.87, 2.18	0.166
TRG	1.50	1.14, 1.96	**0.003**
Size	1.53	0.94, 2.49	0.089
Location	1.17	0.90, 1.53	0.243
Differentiation	1.59	1.12, 2.25	**0.010**
Neoadjuvant	1.15	0.94, 1.41	0.168
ypStage	1.68	1.29, 2.18	** < 0.001**
Step 2			
Age	1.26	0.79, 2.00	0.331
TRG	1.18	0.85, 1.63	0.318
Size	1.21	0.73, 2.03	0.458
Location	1.14	0.88, 1.49	0.314
Differentiation	1.62	1.14, 2.31	**0.007**
Neoadjuvant	1.13	0.93, 1.39	0.219
ypStage	1.26	0.89, 1.78	0.190
Newstage	1.79	1.26, 2.54	**0.001**

^a^*HR* hazard ratio, *CI* confidence interval

## Discussion

This retrospective cohort analysis study found that for patients undergoing curative surgery for gastric cancer after neoadjuvant therapy or conversion therapy, the Newstage system outperformed the AJCC ypTNM system in predicting survival rates. Neoadjuvant therapy has been widely used in the treatment of gastric cancer patients. Karen Becker [10] et al. established a multifactor scoring (PRSC) system based on ypT and ypN from the AJCC TNM staging 6th edition and histopathological tumor regression grade. This system divided patients into three groups with different prognostic outcomes and demonstrated significant predictive efficacy. However, the subsequent TNM staging 8th edition introduced a staging system specifically for patients receiving neoadjuvant therapy. It is important to note that this staging system is based solely on data obtained from the American population. Ziyu Li [11] et al., through analyzing the correlation between survival rates and corresponding ypTNM staging in a Chinese population of 473 gastric cancer patients who received neoadjuvant therapy, demonstrated the effectiveness of the AJCC ypTNM staging system in staging gastric cancer in Asian populations. Furthermore, they found that ypN staging had better prognostic value compared to ypT staging. In their subsequent study [12], they proposed a modified ypTNM staging system based on T staging, N staging, BMI index, and tumor location, and established a predictive model (nomogram) for predicting the prognosis of gastric cancer patients receiving neoadjuvant therapy.

Ian Y.H. Wong [13] et al. proposed a modified ypTNM staging system to predict the prognosis of patients with gastric cancer after neoadjuvant surgery. They reclassified patients with AJCC ypTNM stages I–III based on their survival rates, resulting in the modified ypTNM staging system with stages I, II, IIIA, and IIIB. This system demonstrated significant prognostic differences but did not analyze the patients’ tumor regression grade (TRG). In this study, the Spearman rank correlation coefficient between TRG and ypT is 0.675, indicating a moderate positive correlation between TRG and ypT staging, which is statistically significant (*p* < 0.001). After neoadjuvant therapy, the regression pattern of gastric cancer often exhibits a centrifugal pattern, with tumor cells in the central and superficial regions undergoing necrosis and fibrosis first. Although patients show a good response to chemotherapy and radiation therapy, residual tumor cells are often found in the deeper layers of the tumor bed, which may not necessarily result in a downgrade of the T stage. Previous research [14] has indicated that in over 40% of ypT3 and ypT4 patients, the percentage of residual tumor cells may be less than 10%. However, in ypT1 patients, the percentage of residual tumor cells may not necessarily be lower than 10%. Consistent with the results of this study, several ypT1 patients had a TRG grade of 3 (poor or no response), while ypT3 and ypT4a patients had a TRG grade of 1 (near-complete response). Thus, the correlation analysis between TRG and ypT in this study suggests that TRG can serve as a valuable adjunct to the ypTNM staging system. Jiawang Wei [15] et al. demonstrated that combining ypTNM with TRG can better assess patient prognosis, but they did not incorporate ypN and TRG into the staging system. In this analysis, a Newstage system was developed by combining the 8th AJCC TRG grading with ypN status. The purpose was to assess whether this novel staging method could be used as an effective approach.

although ypTNM and pTNM staging showed overall differences, there were no significant differences in survival rates between pCR, ypT0N + , ypStage I, and ypStage II, making it difficult to visually distinguish between these stages. Subsequently, survival analysis was conducted based on TRG and ypN, revealing that the survival analysis of TRG and ypN could clearly differentiate between the different stages, with ypN showing a distinct separation in survival curves. In the new system, the 3-year overall survival rates were as follows: Newstage I, 94.6%; Newstage II, 79.3%; Newstage III 54.5%; Newstage IV 30.2%. The survival curves demonstrated clear separation and the log-rank test confirmed significant differences between stages I and II (*p* = 0.042) as well as between stages III and IV (*p* = 0.018). Compared to the ypTNM staging system, the new system exhibited superior predictive capability for staging. Furthermore, the calculation of the AIC for the Newstage system and ypTNM staging system incorporated other predictive factors from univariate analysis, which further demonstrated the superiority of the new model. To date, no studies have analyzed the integration of ypN and TRG into the postoperative staging system for gastric cancer patients receiving neoadjuvant therapy.

Both ypT and ypN are independent risk factors that affect disease prognosis. However, ypT alone cannot distinguish the survival rate of patients, possibly because ypT only reflects the tumor stage and does not represent the tumor’s response to neoadjuvant therapy, making it unsuitable for predicting patient prognosis. In contrast, lymph node involvement is significantly associated with poorer survival rates [16]. A meta-analysis of 27 studies found a significant correlation between the proportion of lymph node positivity and worse prognosis [17]. Research by Jian Xian Lin [18] suggests that postoperative adjuvant chemotherapy is associated with a lymph node positivity rate of 9% or higher, which can improve prognosis. Additionally, Fujitani’s study [19] also confirmed ypN as an independent prognostic factor. ypN is also influenced by factors such as response to neoadjuvant therapy and the location of the lesion, making it unreliable to rely solely on ypT and ypN for staging systems. Tumor Regression Grade (TRG) is an indicator used to assess the extent of pathological response of tumors after neoadjuvant therapy. Currently, there are nine different TRG grading systems, and there is no universally recognized standard [20]. In gastric cancer, the TRG grading system of the AJCC 8th edition is commonly used, with some studies also using the Becker or Mandard criteria. Studies [21] have shown that tumor histological regression after chemotherapy is an important prognostic factor for gastrointestinal tumors and has a greater impact on patient prognosis than tumor depth of invasion (ypT staging). Therefore, it is reasonable to establish a prognostic staging system combining TRG and ypN to evaluate patient prognosis. Similar explorations [22] are also being conducted in esophageal cancer. By randomly combining TRG and ypN, a new staging system is formed, which has better predictive efficacy for patient prognosis compared to the ypTNM staging system. In the studies by A.R. Davies [23] and Takaomi Hagi [24] on neoadjuvant therapy for esophageal cancer, grading of lymph node tumor regression was confirmed to predict long-term survival rates in patients.

The patients were divided into two groups based on the years 2015–2018 and 2019–2020, with 83 cases in the 2015–2018 group and 175 cases in the 2019–2020 group. The 5-year survival rate for patients in the 2015–2018 group was 63.9%, with a median follow-up time of 74 months. However, due to the limited number of cases in the 2015–2018 group, which may be attributed to the relatively late adoption of neoadjuvant treatment in our country leading to fewer patients undergoing this treatment, and the fact that the 2019–2020 group has not yet reached the 5-year survival time, a comprehensive analysis of the 5-year survival rate for the entire cohort was not conducted, leading to some uncertainty. We also conducted survival analysis for the two groups based on the Newstage system. From the survival curves, it can be observed that Newstage I, II, III, and IV still exhibit a distinct trend (Supplementary Figure S4A, B). Therefore, we look forward to further refining the 5-year survival rate for patients in this cohort in the future to further demonstrate the effectiveness of the Newstage system.

Based on the preoperative imaging data, including CT, MRI, and PET-CT, neoadjuvant chemotherapy is recommended for clinical staging of cT1-2N1-3M0, cT3-4N1-3M0 for esophageal and gastric junction cancers, as well as cT3-4aN + M0 for non-esophageal and gastric junction cancers. Subsequently, after internal departmental or multidisciplinary discussions, the specific neoadjuvant chemotherapy regimen for the patient is determined. In this retrospective analysis, the proportion of patients receiving neoadjuvant therapy early on was relatively small and no selection was made on the neoadjuvant chemotherapy regimen. Some patients already adopted the SOX regimen. However, in 2019, there was an increase in the number of patients receiving neoadjuvant therapy, and as a result, the SOX and XELOX regimens started to be widely used. Therefore, it was difficult to determine the impact of the RESOLVE study on clinical practice at that time. The emergence of neoadjuvant therapy for gastric cancer began after the publication of the RESOLVE study results. Currently, neoadjuvant combined with immunotherapy, targeted therapy, and other regimens have shown good efficacy [25], including PRODIGY [26], PETRARCA [27], GERCOR NEONIPIGA [28], and others. A survival analysis of the 2015–2018 and 2019–2020 groups revealed no significant differences between the two groups (Supplementary Figure S4C). We believe that despite differences in neoadjuvant treatment modalities and regimens during different time periods, T stage, N stage, and TRG grading remain the primary factors affecting prognosis. The impact of adjustments in neoadjuvant treatment modalities is primarily reflected in the changes in staging. The Newstage system is based on TRG grading and ypN staging and thus is applicable to gastric cancer patients receiving different neoadjuvant treatment regimens. We also look forward to prospective studies on different neoadjuvant treatment regimens to further validate the effectiveness of the Newstage system.

In this study, the postoperative adjuvant chemotherapy regimen was missing for most patients. Further analysis and exploration are needed to determine whether the choice of postoperative adjuvant chemotherapy regimen has an impact on prognosis. New clinical trials are still ongoing, and therefore, the classification of the Newstage system also requires continuous exploration and improvement. It is worth noting that the studies mentioned above did not include patients in stage M1, which represents distant organ or site metastasis in gastric cancer. However, in M1 stage, the extent and number of metastatic lesions are often limited or relatively few, known as "oligometastasis". These patients, after undergoing aggressive conversion therapy and achieving R0 resection, have a favorable prognosis [29]. In future studies, it may be considered to include them in the analysis.

The neoadjuvant therapy methods for gastric cancer are continuously advancing, including immunotherapy and targeted therapy. In China, the commonly used chemotherapy regimens are SOX and XELOX, while the use of combined immunotherapy is relatively limited. Further exploration is needed to determine the impact of these treatment methods on patient survival rates. In the era of precision medicine, grading lymph node regression to improve the accuracy of staging is beneficial for enhancing treatment outcomes. Ongoing research in this area aims to optimize the treatment strategies for gastric cancer.

This study has several limitations. Firstly, it is a single-center retrospective study, which precludes randomization and intervention. It relies solely on clinical data, raising the risk of data bias. Secondly, the time span of the included cases is less than 5 years, which prevents the analysis of 5-year overall survival. Further analysis should be conducted in future studies to address this limitation. Thirdly, the sample size of the single-center study is limited, particularly for cases with TRG grade 0 and N stage 1, 2, or 3, which may impact the staging results. Additionally, a notable drawback of this retrospective analysis study is the absence of explicit criteria for the selection of different neoadjuvant treatment regimens. Therefore, in order to apply the research findings to clinical practice, larger sample sizes, and multicenter validation are necessary.

## Conclusion

The establishment of a new staging system, the Newstage system, based on the combination of TRG and ypN, holds great promise in terms of its application potential. It has significant clinical implications for guiding clinical decisions, developing individualized treatment plans, and assessing patient prognosis in gastric cancer. However, further research and validation are still necessary to confirm these findings and promote their application in clinical practice.

### Supplementary Information

**Additional file 1: Supplementary Table S1.** Comparison of 3-year overall survival for pCR, ypT0 N + and AJCC ypTNM substages I-III. **Supplementary Table S2.** Comparison of 3-year overall survival for pCR, pT0N + and AJCC pTNM substages IA–III. **Supplementary Table S3.** 3y-Overall survival for the combination of TRG and ypN. **Supplementary Table S4.** Comparison of 3-year overall survival for Newstage system I-IV. **Supplementary Table S5.** Univariate analysis for survival using Cox regression analysis on cohort. **Supplementary Figure S1.** Kaplan–Meier overall survival according to the 8th AJCC pTNM stage and ypN status. Kaplan–Meier overall survival according to the 8th AJCC pTNM stage(A), ypN status(B). **Supplementary Figure S2.** Kaplan–Meier overall survival according to the combination of TRG and ypN status. **Supplementary Figure S3.** Bootstrap average ROC curve. Internal validation using the Bootstrap method with 2000 resamples further supported the superiority of the Newstage system, as evidenced by the mean AUC of 0.756. **Supplementary Figure S4.** Effect of the years 2015–2018 and 2019–2020 groups on overall survival. Kaplan–Meier overall survival stratified for the Newstage for 2015–2018 patients (A) and 2019–2020 patiens (B), and Kaplan–Meier survival curves for all patients according to the year of operation (C).

## Data Availability

The data in this study are available from the author for correspondence upon reasonable request.

## References

[1] Sung H, Ferlay J, Siegel RL (2021). Global cancer statistics 2020: GLOBOCAN estimates of incidence and mortality worldwide for 36 cancers in 185 countries. CA Cancer J Clin.

[2] Zheng R, Zhang S, Zeng H (2022). Cancer incidence and mortality in China, 2016. J Natl Cancer Cent.

[3] Cunningham D. Perioperative chemotherapy versus surgery alone for resectable gastroesophageal cancer. N Engl J Med. 2006;355:11–20. 10.1056/NEJMoa055531. 10.1056/NEJMoa055531 16822992

[4] Noh SH, Park SR, Yang H-K (2014). Adjuvant capecitabine plus oxaliplatin for gastric cancer after D2 gastrectomy (CLASSIC): 5-year follow-up of an open-label, randomised phase 3 trial. Lancet Oncol.

[5] Zhang X, Liang H, Li Z (2021). Perioperative or postoperative adjuvant oxaliplatin with S-1 versus adjuvant oxaliplatin with capecitabine in patients with locally advanced gastric or gastro-oesophageal junction adenocarcinoma undergoing D2 gastrectomy (RESOLVE): an open-label, superiority and non-inferiority, phase 3 randomised controlled trial. Lancet Oncol.

[6] In H, Ravetch E, Langdon-Embry M (2018). The newly proposed clinical and post-neoadjuvant treatment staging classifications for gastric adenocarcinoma for the American Joint Committee on Cancer (AJCC) staging. Gastric Cancer Off J Int Gastric Cancer Assoc Jpn Gastric Cancer Assoc.

[7] Smyth EC, Fassan M, Cunningham D (2016). Effect of pathologic tumor response and nodal status on survival in the medical research council adjuvant gastric infusional chemotherapy trial. J Clin Oncol.

[8] Schmidt T, Sicic L, Blank S (2014). Prognostic value of histopathological regression in 850 neoadjuvantly treated oesophagogastric adenocarcinomas. Br J Cancer.

[9] Amin MB, Greene FL, Edge SB (2017). The Eighth Edition AJCC Cancer Staging Manual: Continuing to build a bridge from a population-based to a more “personalized” approach to cancer staging. CA Cancer J Clin..

[10] Becker K, Reim D, Novotny A (2012). Proposal for a multifactorial prognostic score that accurately classifies 3 groups of gastric carcinoma patients with different outcomes after neoadjuvant chemotherapy and surgery. Ann Surg.

[11] Li Z, Wang Y, Shan F (2018). ypTNM staging after neoadjuvant chemotherapy in the Chinese gastric cancer population: an evaluation on the prognostic value of the AJCC eighth edition cancer staging system. Gastric Cancer Off J Int Gastric Cancer Assoc Jpn Gastric Cancer Assoc.

[12] Li Z, Xiao Q, Wang Y (2020). A modified ypTNM staging system–development and external validation of a nomogram predicting the overall survival of gastric cancer patients received neoadjuvant chemotherapy. Cancer Manag Res.

[13] Lin JX, Yoon C, Desiderio J (2019). Development and validation of a staging system for gastric adenocarcinoma after neoadjuvant chemotherapy and gastrectomy with D2 lymphadenectomy. Br J Surg.

[14] Ott K, Blank S, Becker K (2013). Factors predicting prognosis and recurrence in patients with esophago-gastric adenocarcinoma and histopathological response with less than 10 % residual tumor. Langenbecks Arch Surg.

[15] Wei J, Huang R, Guo S (2018). ypTNM category combined with AJCC tumor regression grade for screening patients with the worst prognosis after neoadjuvant chemoradiation therapy for locally advanced rectal cancer. Cancer Manag Res.

[16] Woodham BL, Chmelo J, Donohoe CL (2020). Prognostic significance of lymphatic, venous and perineural invasion after neoadjuvant chemotherapy in patients with gastric adenocarcinoma. Ann Surg Oncol.

[17] Zhu J, Xue Z, Zhang S (2018). Integrated analysis of the prognostic role of the lymph node ratio in node-positive gastric cancer: a meta-analysis. Int J Surg.

[18] Lin J-X, Tang Y-H, Lin G-J (2022). Association of adjuvant chemotherapy with overall survival among patients with locally advanced gastric cancer after neoadjuvant chemotherapy. JAMA Netw Open.

[19] Fujitani K, Nakamura K, Mizusawa J (2021). Posttherapy topographical nodal status, ypN-site, predicts survival of patients who received neoadjuvant chemotherapy followed by curative surgical resection for non-type 4 locally advanced gastric cancer: supplementary analysis of JCOG1004-a. Gastric Cancer.

[20] Klevebro F, Tsekrekos A, Low D (2020). Relevant issues in tumor regression grading of histopathological response to neoadjuvant treatment in adenocarcinomas of the esophagus and gastroesophageal junction. Dis Esophagus Off J Int Soc Dis Esophagus..

[21] Becker K, Langer R, Reim D (2011). Significance of histopathological tumor regression after neoadjuvant chemotherapy in gastric adenocarcinomas: a summary of 480 cases. Ann Surg.

[22] Wong IYH, Chung JCY, Zhang RQ (2022). A novel tumor staging system incorporating tumor regression grade (TRG) with lymph node status (ypN-category) results in better prognostication than ypTNM stage groups after neoadjuvant therapy for esophageal squamous cell carcinoma. Ann Surg.

[23] Davies AR, Myoteri D, Zylstra J (2018). Lymph node regression and survival following neoadjuvant chemotherapy in oesophageal adenocarcinoma. Br J Surg.

[24] Hagi T, Makino T, Yamasaki M (2022). Pathological regression of lymph nodes better predicts long-term survival in esophageal cancer patients undergoing neoadjuvant chemotherapy followed by surgery. Ann Surg.

[25] Joshi S, Badgwell BD (2021). Current treatment and recent progress in gastric cancer. CA Cancer J Clin.

[26] Kang Y-K, Yook JH, Park Y-K (2021). PRODIGY: a phase III study of neoadjuvant docetaxel, oxaliplatin, and S-1 plus surgery and adjuvant S-1 versus surgery and adjuvant S-1 for resectable advanced gastric cancer. J Clin Oncol.

[27] FLOT versus FLOT/trastuzumab/pertuzumab perioperative therapy of human epidermal growth factor receptor 2-positive resectable esophagogastric adenocarcinoma: a randomized phase II trial of the AIO EGA Study Group - PubMed [Internet]. [cited 2023 Sep 13]. Available from: https://pubmed.ncbi.nlm.nih.gov/35709415/. 10.1200/JCO.22.00380 35709415

[28] André T, Tougeron D, Piessen G (2023). Neoadjuvant nivolumab plus ipilimumab and adjuvant nivolumab in localized deficient mismatch repair/microsatellite instability-high gastric or esophagogastric junction adenocarcinoma: the GERCOR NEONIPIGA phase II study. J Clin Oncol Off J Am Soc Clin Oncol.

[29] Fukuchi M, Ishiguro T, Ogata K (2015). Prognostic role of conversion surgery for unresectable gastric cancer. Ann Surg Oncol.

