# Supplementary material for: Development and evaluation of the Newstage system: integrating tumor regression grade and lymph node status for improved prognostication in neoadjuvant treatment of gastric cancer
Source: World J Surg Oncol. 2024 Jan 10;22:16. doi: 10.1186/s12957-023-03291-4 (PMC10777530; doi:10.1186/s12957-023-03291-4)
Supplement: Supplementary file 1 — Additional file 1: Supplementary Table S1. Comparison of 3-year overall survival for pCR, ypT0 N + and AJCC ypTNM substages I-III. Supplementary Table S2. Comparison of 3-year overall survival for pCR, pT0N + and AJCC pTNM substages IA–III. Supplementary Table S3. 3y-Overall survival for the combination of TRG and ypN. Supplementary Table S4. Comparison of 3-year overall survival for Newstage system I-IV. Supplementary Table S5. Univariate analysis for survival using Cox regression analysis on cohort. Supplementary Figure S1. Kaplan–Meier overall survival according to the 8th AJCC pTNM stage and ypN status. Kaplan–Meier overall survival according to the 8th AJCC pTNM stage(A), ypN status(B). Supplementary Figure S2. Kaplan–Meier overall survival according to the combination of TRG and ypN status. Supplementary Figure S3. Bootstrap average ROC curve. Internal validation using the Bootstrap method with 2000 resamples further supported the superiority of the Newstage system, as evidenced by the mean AUC of 0.756. Supplementary Figure S4. Effect of the years 2015–2018 and 2019–2020 groups on overall survival. Kaplan–Meier overall survival stratified for the Newstage for 2015–2018 patients (A) and 2019–2020 patiens (B), and Kaplan–Meier survival curves for all patients according to the year of operation (C). [file 12957_2023_3291_MOESM1_ESM.docx]

**Development and Evaluation of the Newstage System: Integrating Tumor Regression Grade and Lymph Node Status for Improved Prognostication in Neoadjuvant Treatment of Gastric Cancer**

Ming Chen^1^†, Shanshan Yu^1^†, Cheng Chen^1^†, Jinxiao Liang^1^, Donghui Zhou^1^*

1Department of Surgical Oncology, The First Affiliated Hospital, School of Medicine, Zhejiang University, Hangzhou, China

*Correspondence: M.D, Professor Donghui Zhou, Department of Surgical Oncology, The First Affiliated Hospital, School of Medicine, Zhejiang University, Hangzhou, China. Phone number: 0571-87236878, E-mail: 1193076@zju.edu.cn.

**Table S1** Comparison of 3-year overall survival for pCR, ypT0 N+ and AJCC ypTNM substages I-III

|  | 3-year OS (%) | pCR | ypT0N+ | ypStage I | ypStage II | ypStage III |
| --- | --- | --- | --- | --- | --- | --- |
| pCR | 93.8 | - | **0.438** | **0.354** | 0.011 | <0.001 |
| ypT0N+ | 100.0 | **0.438** | - | **0.349** | **0.151** | 0.017 |
| ypStage I | 85.3 | **0.354** | **0.349** | - | 0.013 | <0.001 |
| ypStage II | 69.3 | 0.011 | **0.151** | 0.013 | - | <0.001 |
| ypStage III | 39.7 | <0.001 | 0.017 | <0.001 | <0.001 | - |

Data in stage-labelled columns are *P* values.

**Table S2** Comparison of 3-year overall survival for pCR, pT0N+ and AJCC pTNM substages IA–IIIC

|  | 3-year OS (%) | pCR | pT0N+ | pStage IA | pStage IB | pStage IIA | pStage IIB | pStage IIIA | pStage IIIB | pStage IIIC |
| --- | --- | --- | --- | --- | --- | --- | --- | --- | --- | --- |
| pCR | 93.8 | - | **0.438** | **0.427** | **0.196** | 0.038 | 0.003 | <0.001 | <0.001 | <0.001 |
| pT0N+ | 100.0 | **0.438** | - | **0.364** | **0.277** | **0.191** | **0.090** | **0.052** | 0.012 | 0.002 |
| pStage IA | 86.4 | **0.427** | **0.364** | - | **0.575** | **0.090** | 0.004 | <0.001 | <0.001 | <0.001 |
| pStage IB | 77.8 | **0.196** | **0.277** | **0.575** | **-** | **0.708** | **0.368** | **0.144** | 0.018 | 0.001 |
| pStage IIA | 71.8 | 0.038 | **0.191** | **0.090** | **0.708** | - | **0.342** | 0.053 | <0.001 | <0.001 |
| pStage IIB | 69.2 | 0.003 | **0.090** | 0.004 | **0.368** | **0.342** | - | **0.262** | 0.004 | <0.001 |
| pStage IIIA | 50.0 | <0.001 | **0.052** | <0.001 | **0.144** | **0.053** | **0.262** | - | 0.047 | <0.001 |
| pStage IIIB | 32.0 | <0.001 | 0.012 | <0.001 | 0.018 | <0.001 | 0.004 | 0.047 | - | 0.049 |
| pStage IIIC | 8.33 | <0.001 | 0.002 | <0.001 | 0.001 | <0.001 | <0.001 | <0.001 | 0.049 | - |

Data in stage-labelled columns are *P* values.

**Table S3 3y-**Overall survival for combination of TRG and ypN

| ypN TRG | 0 | 1 | 2 | 3a | 3b |
| --- | --- | --- | --- | --- | --- |
| 0 | 0.938 | 1 | 1 | - | - |
| 1 | 0.795 | 0.75 | 0.667 | - | 0.5 |
| 2 | 0.763 | 0.791 | 0.500 | 0.364 | 0 |
| 3 | 0.833 | 0.556 | 0.444 | 0.286 | 0 |

Data in columns are 3y-overall survival rate.

**Table S4** Comparison of 3-year overall survival for Newstage system I-IV

|  | 3-year OS (%) | New I | New II | New III | New IV |
| --- | --- | --- | --- | --- | --- |
| New I | 94.6 | - | 0.042 | <0.001 | <0.001 |
| New II | 79.3 | 0.042 | - | <0.001 | <0.001 |
| New III | 54.5 | <0.001 | <0.001 | - | 0.018 |
| New IV | 30.2 | <0.001 | <0.001 | 0.018 | - |

Data in stage-labelled columns are *P* values.

**Table S5** Univariate analysis for survival using Cox regression analysis on cohort

|  |  | **univariate** | | |
| --- | --- | --- | --- | --- |
| **Characteristic** | **N(%)** | **HR**^1^ | **95% CI**^1^ | **p-value** |
| **Gender** |  |  |  | 0.771 |
| F | 78(30.2%) | — | — |  |
| M | 180(69.8%) | 1.07 | 0.68, 1.69 | 0.771 |
| **Age** |  |  |  | 0.909 |
| ≤60 | 85(32.9%) | — | — |  |
| >60 | 173(67.1%) | 1.03 | 0.66, 1.60 | 0.909 |
| **TRG** |  |  |  | **<0.001** |
| 0 | 37(14.3%) | — | — |  |
| 1 | 48(18.6%) | 2.83 | 0.78, 10.3 | 0.114 |
| 2 | 88(34.1%) | 6.08 | 1.87, 19.8 | 0.003 |
| 3 | 85(33.0%) | 8.22 | 2.55, 26.5 | <0.001 |
| **ypStage** |  |  |  | **<0.001** |
| pCR | 28(10.9%) | — | — |  |
| ypT0N+ | 5(1.9%) | 0.00 | 0.00, Inf | 0.995 |
| ypStage I | 71(27.5%) | 1.79 | 0.50, 6.42 | 0.371 |
| ypStage II | 76(29.5%) | 4.28 | 1.29, 14.2 | 0.017 |
| ypStage III | 78(30.2%) | 10.7 | 3.34, 34.4 | <0.001 |
| **Size(cm)** |  |  |  | **<0.001** |
| ≤5 | 211(81.8%) | — | — |  |
| >5 | 47(18.2%) | 2.81 | 1.79, 4.39 | <0.001 |
| **Location** |  |  |  | 0.442 |
| Diffuse | 6(2.3%) | — | — |  |
| Lower third | 130(50.4%) | 0.27 | 0.10, 0.75 | 0.012 |
| Middle third | 74(28.7%) | 0.38 | 0.13, 1.08 | 0.069 |
| Upper third | 48(18.6%) | 0.22 | 0.07, 0.68 | 0.009 |
| **Differentiation** |  |  |  | 0.294 |
| High | 7(2.7%) | — | — |  |
| Middle | 95(36.8%) | 21,112,524 | 0.00, Inf | 0.995 |
| Poor | 126(48.9%) | 36,075,914 | 0.00, Inf | 0.995 |
| Unknown | 30(11.6%) | 13,552,897 | 0.00, Inf | 0.995 |
| **Neoadjuvant** |  |  |  | **0.037** |
| SOX | 176(68.2%) | — | — |  |
| XELOX | 46(17.8%) | 1.67 | 1.00, 2.80 | 0.051 |
| FOLFOX | 21(8.2%) | 2.16 | 1.15, 4.04 | 0.017 |
| ELSE | 15(5.8%) | 1.33 | 0.57, 3.10 | 0.506 |
| **Combined immunotherapy** |  |  |  | 0.250 |
| Yes | 46(17.8%) | — | — |  |
| No | 212(81.2%) | 0.74 | 0.45, 1.23 | 0.250 |
| **Post-operative adjuvant therapy** |  |  |  | 0.846 |
| Yes | 222(86.0%) | — | — |  |
| No | 36(14.0%) | 0.94 | 0.51, 1.73 | 0.846 |
| **Newstage** |  |  |  | **<0.001** |
| I | 37(14.3%) | — | — |  |
| II | 135(52.3%) | 3.19 | 0.97, 10.4 | 0.055 |
| III | 33(12.8%) | 8.45 | 2.49, 28.7 | <0.001 |
| IV | 53(20.6%) | 16.0 | 4.93, 52.1 | <0.001 |

**^1^HR = Hazard Ratio, CI = Confidence Interval**

**Figure S1** Kaplan-Meier overall survival according to the 8th AJCC pTNM stage and ypN status.

**
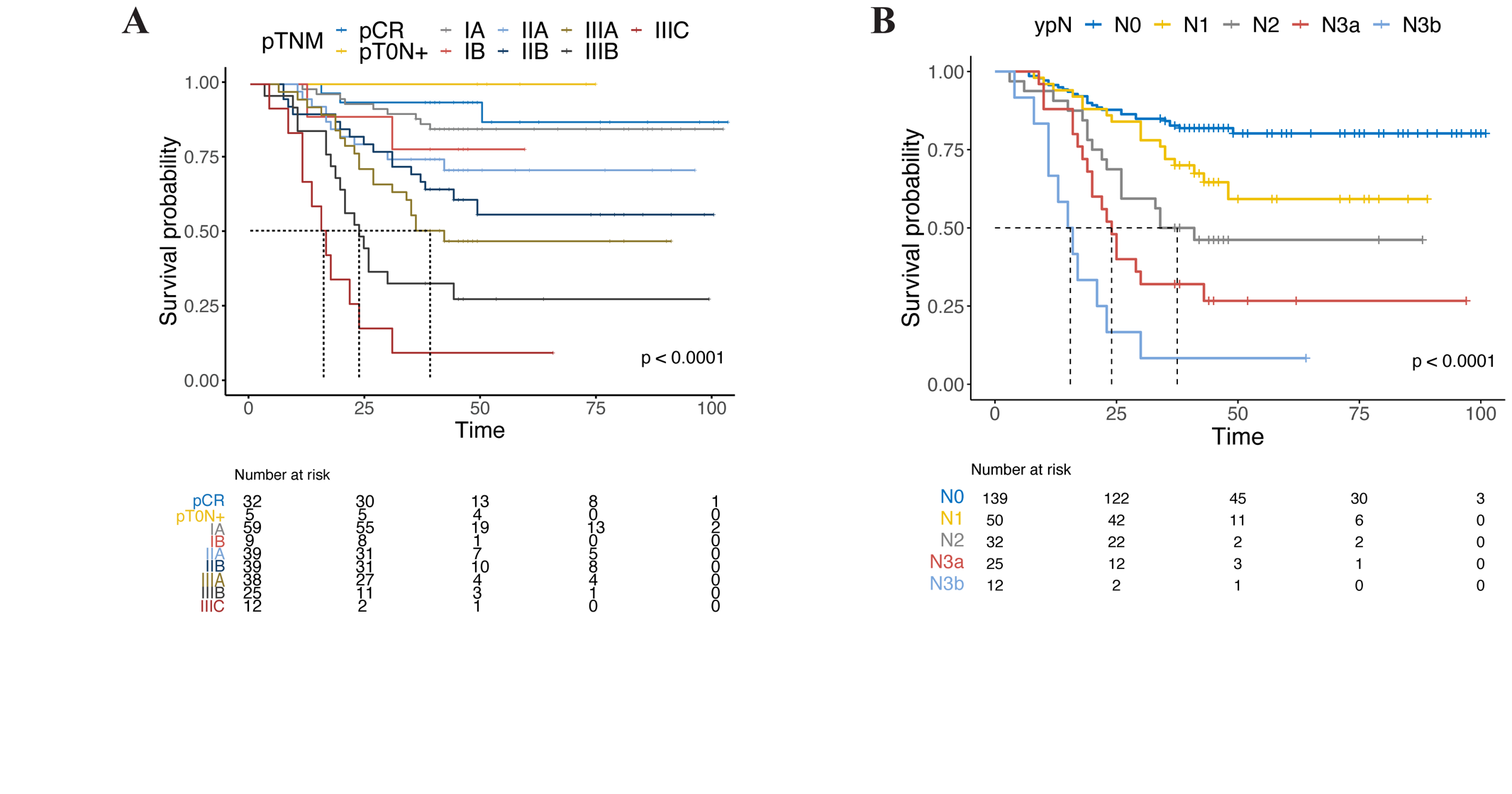
**

Kaplan-Meier overall survival according to the 8th AJCC pTNM stage(A), ypN status(B).

**Figure S2** Kaplan-Meier overall survival according to the combination of TRG and ypN status.

**
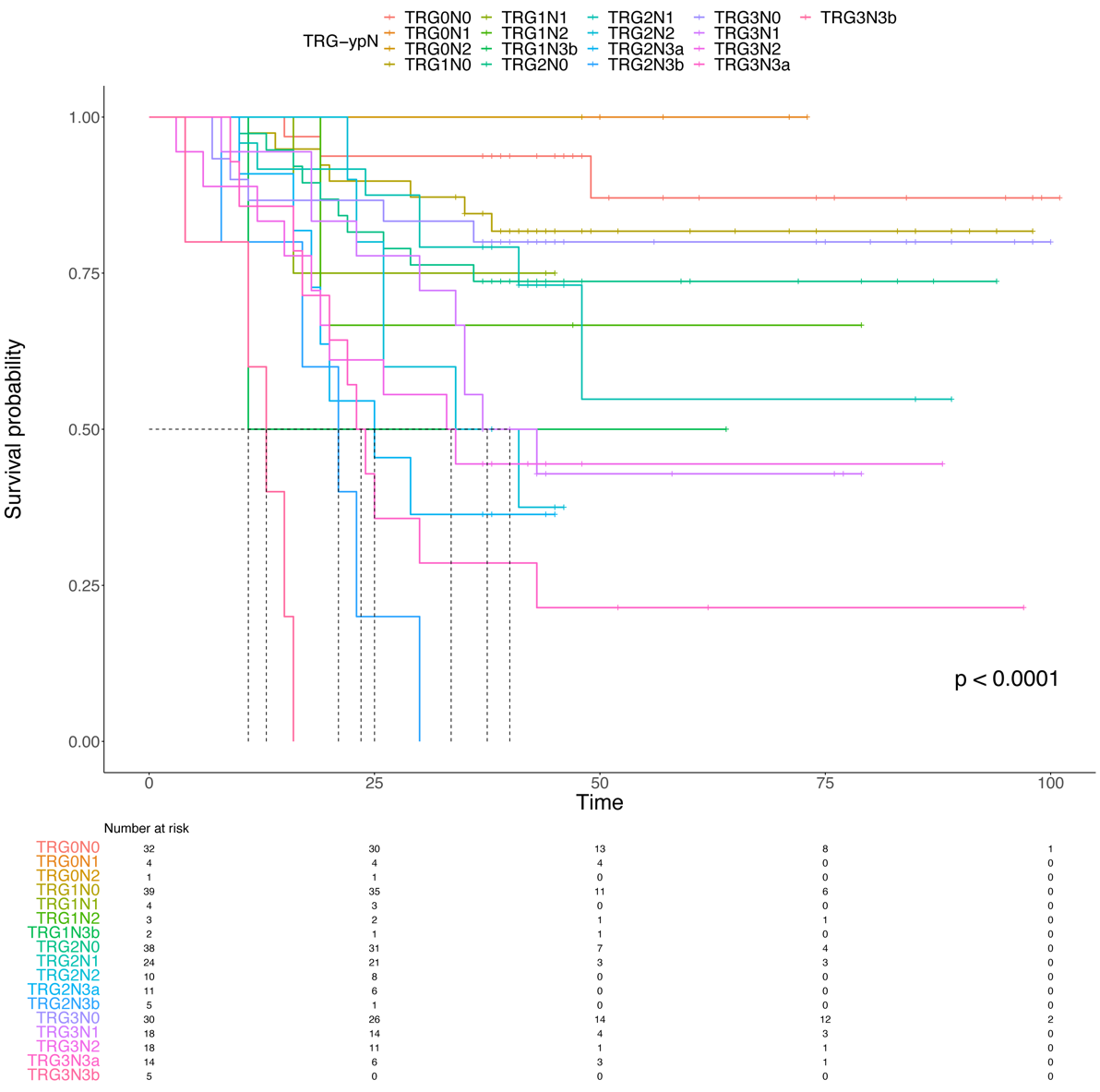
**

**Figure S3** Bootstrap average ROC curve


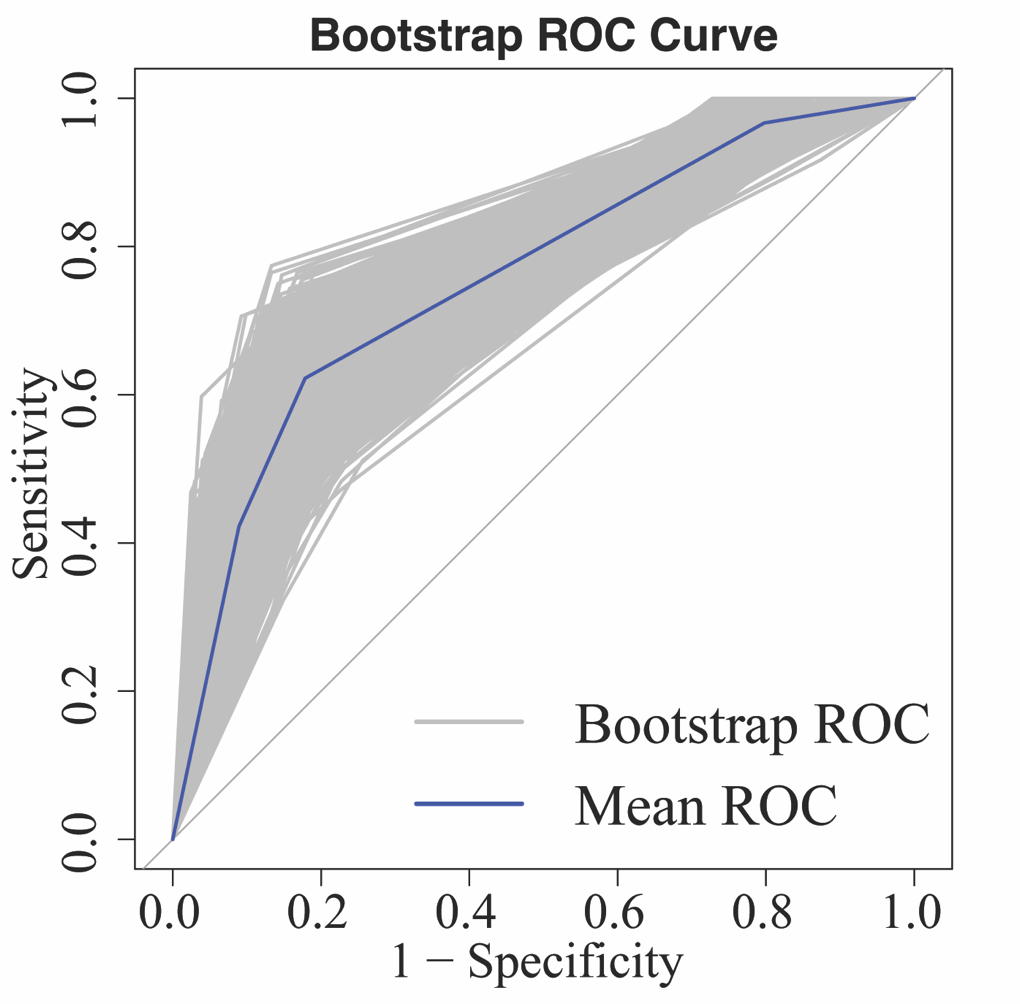


**Figure S4** Effect of the years 2015-2018 and 2019-2020 groups on overall survival.


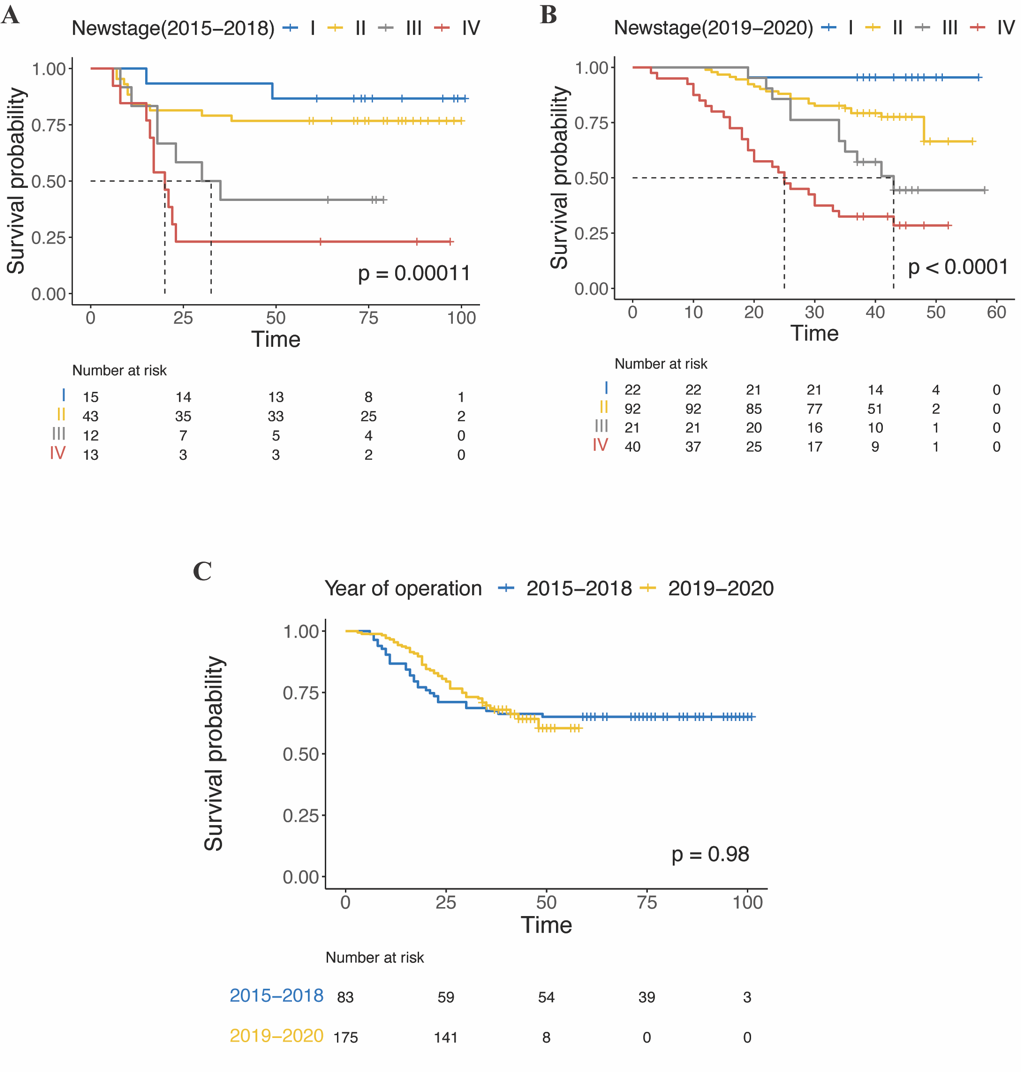


Kaplan–Meier overall survival stratified for the Newstage for 2015-2018 patients (A) and 2019-2020 patiens (B), and Kaplan-Meier survival curves for all patients according to the year of operation (C).
